# Influence of Free Fatty Acids on Lipid Membrane–Nisin Interaction

**DOI:** 10.1021/acs.langmuir.0c02266

**Published:** 2020-11-02

**Authors:** Francesca Saitta, Paolo Motta, Alberto Barbiroli, Marco Signorelli, Carmelo La Rosa, Anna Janaszewska, Barbara Klajnert-Maculewicz, Dimitrios Fessas

**Affiliations:** † Dipartimento di Scienze per gli Alimenti, la Nutrizione e l’Ambiente, DeFENS, Università degli Studi di Milano, Via Celoria 2, 20133, Milano, Italy; ‡ Department of General Biophysics, Faculty of Biology and Environmental Protection, University of Lodz, 141/143 Pomorska Street, 90-236 Lodz, Poland; § Dipartimento di Scienze Chimiche, Università degli Studi di Catania, Viale Andrea Doria 6, 95125, Catania, Italy

## Abstract

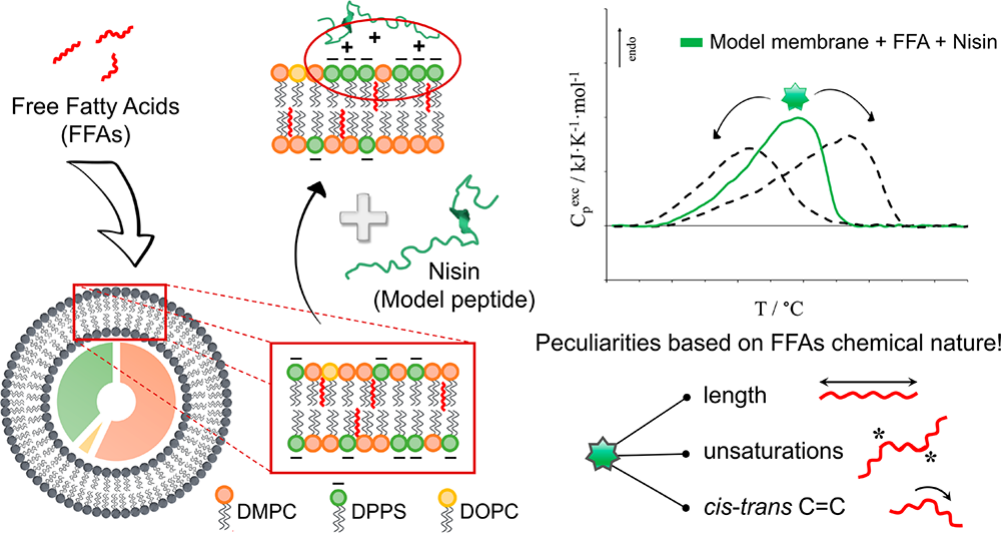

The influence of free fatty acids (FFAs) on the nisin–membrane interaction was investigated through micro-DSC and fluorescence spectroscopy. A simple but informative model membrane was prepared (5.7 DMPC:3.8 DPPS:0.5 DOPC molar ratio) by considering the presence of different phospholipid headgroups in charge and size and different phospholipid tails in length and unsaturation level, allowing the discrimination of the combined interaction of nisin and FFAs with the single phospholipid constituents. The effects of six FFAs on membrane stability were evaluated, namely two saturated FFAs (palmitic acid and stearic acid), two monounsaturated FFAs (*cis*-unsaturated oleic acid and *trans*-unsaturated elaidic acid) and two *cis*-polyunsaturated FFAs (ω-6 linoleic acid and ω-3 docosahexaenoic acid). The results permitted assessment of a thermodynamic picture of such interactions which indicates that the peptide–membrane interaction does not overlook the presence of FFAs within the lipid bilayer since both FFAs and nisin are able to selectively promote thermodynamic phase separations as well as a general lipid reorganization within the host membrane. Furthermore, the magnitude of the effects may be different depending on the FFA chemical structure as well as the membrane lipid composition.

## Introduction

Fatty acids can be naturally found in several foods, such as nuts, seeds, fruits, dairy products, vegetable oils, fish oils, and animal fats, but they can also be obtained through food processing.^1−3^ They have been extensively studied because of their links to nutrition and health.^4,5^ Indeed, fatty acids have been associated with several benefits or pathologies depending on their chemical structures. For instance, *cis*-unsaturated fatty acids have received much attention as antimicrobials^6,7^ and therapeutics,^8−11^ with particular consideration for polyunsaturated fatty acids since humans cannot synthesize them. By contrast, saturated and *trans*-unsaturated fatty acids have often been shown to play relevant roles in the onset and progression of several diseases.^12−15^

Beside the overall effects that fatty acids may have on health, the molecular action of the free fatty acids (FFAs) fraction has also gained much interest in past decades because of their common biological functions. Indeed, FFAs have been shown to be involved in several membrane-mediated cellular processes, from the modulation of signaling processes and membrane-bound protein functions to the fusion of lipid vesicles and cells and/or modification of lipid microdomains of cell phospholipid bilayers.^7,8,16−19^ However, though low percentages of FFAs are naturally present in plasma and in cell membranes (around 0.3–10% of total lipids),^20^ altered levels of FFAs are recurrent in pathological cases (e.g., diabetic and/or obese subjects) and may cause strong alterations in cell metabolism^21−23^ as well as in cell phospholipid bilayers’ physicochemical properties and thermodynamics.^24,25^

Recently, multistep calorimetric studies on the influence of FFAs on the thermodynamic stabilities of several liposomes considered as model cell membranes were performed;^25,26^ also considered were the several factors that may affect the physicochemical behaviors of real cell membranes (size, lipid composition, presence of cholesterol, etc.) following the insulin secretory granules’ membrane paradigm.^27^ Indeed, the thermotropic behaviors of phospholipid vesicles and membranes have been shown to be severely influenced by the lipid composition as well as by the interaction of the bilayer with external agents.^28−34^

In this framework, this study was focused on the effects that the presence of FFAs may have on peptide–membrane interaction. Indeed, despite the literature report studies on peptide/protein interaction with membranes,^35−38^ to our knowledge no evidence is reported about the possible influence that FFAs-derived modifications in membrane thermodynamics may have on the interaction of cell membrane phospholipid bilayers with external molecules as peptides and/or proteins.

For this purpose, we selected a model peptide that followed two main characteristics suitable for this FFAs–peptide–membrane interaction study, i.e., the ability of directly interacting with phospholipid membranes and the simplicity of the tertiary structure to avoid superimposed phenomena to the membrane phase transition. Nisin, a small cationic peptide known for its ability to interact with cell membranes both directly and by a receptor-mediated way,^38,39^ was in line with the listed criteria and, therefore, was selected as a model in this work. Furthermore, on the basis of the thermodynamic information achieved from previous studies,^25,26^ a model cell membrane was designed and prepared as a reference liposome system by combining specific percentages of DMPC, DPPS, and DOPC in order to consider the main compositional aspects (phospholipid headgroup, tails, presence of unsaturations) and to resemble the thermal stability profile commonly observed in both real cell membranes and highly representative artificial ones in terms of cooperativity and enthalpy contributions to the gel-to-liquid-crystalline phase transition.^26,40,41^ Nisin–vesicle interaction was investigated through micro-DSC and fluorescence spectroscopy in FFAs-free and FFAs-containing liposomes at physiological pH (pH 7.4). The addition of 20% FFAs was considered in order to simulate pathological conditions as well as to enhance the alterations on the thermograms and better appreciate the phenomena involved. The effects of six different FFAs on membrane stability were evaluated, namely, two saturated FFAs (palmitic acid and stearic acid), two monounsaturated FFAs (the *cis*-unsaturated oleic acid and the *trans*-unsaturated elaidic acid), and two *cis*-polyunsaturated FFAs (the ω-6 linoleic acid and the ω-3 docosahexaenoic acid or DHA).

## Experimental Section

### Materials

1,2-Dimyristoyl-*sn*-glycero-3-phosphocholine (DMPC), 1,2-dioleoyl-*sn*-glycero-3-phosphocholine (DOPC), and 1,2-dipalmitoyl-*sn*-glycero-3-phospho-l-serine (DPPS, sodium salt) powders were purchased from Avanti Polar Lipids (purity certified by the supplier >99%), whereas palmitic acid (PA), stearic acid (SA), oleic acid (OA), elaidic acid (EA), linoleic acid (LA), and docosahexaenoic acid (DHA), as well as nisin (lyophilized powder containing ∼2.5% w/w nisin), solvents, and the other chemicals were obtained from Sigma-Aldrich. The lipids were of the highest available purity (≥99%) and were used without further purification. All solvents were of analytical grade.

### Nisin Purification and Preparation of Stock Solutions

Nisin was purified following a procedure described elsewhere.^42^ In brief, commercial nisin (N5764, lyophilized powder containing ∼2.5% w/w nisin, Sigma-Aldrich) was dissolved 1.3 g/100 mL in 50 mM sodium lactate acid, pH 3. The nisin solution was filtered through 0.45 μm pores and applied to a 5 mL SP Sepharose fast flow cation exchange column (Sigma-Aldrich). After a washing step with 50 mL of 600 mM NaCl, purified nisin was eluted from the column with the use of 50 mL of 800 mM NaCl. To remove NaCl, protein in the elution fractions was precipitated with 20% (v/v) trichloroacetic acid (TCA) overnight at 4 °C. Precipitated protein was washed twice with ice-cold acetone to remove residual TCA. The purity grade of nisin was quantified by HPLC, revealing a purity grade of >95% (Figures S1 and S2).

Nisin stock solutions were daily prepared by dissolution of the appropriate protein amounts in 10 mM phosphate buffer (pH 7.4). In order to help the protein solubilization, nisin solutions were subjected to a mild sonication until clear samples were obtained.^43^

### Liposome Preparation

Liposomes were prepared through thin-film hydration.^44^ Phospholipids were dissolved in chloroform:methanol 3:1 in a round-bottomed flask, dried under a stream of dry nitrogen gas, and evaporated to dryness through rotary evaporation (Heidolph Laborota 4000 efficient, WB eco, Schwabach, Germany) at 45 °C. The films were kept under vacuum for at least 3 h to remove solvent traces and then aged overnight at 4 °C. For the hydration, 10 mM phosphate buffer (pH 7.4) at a temperature above the gel-to-liquid-crystalline transition of the lipid system was added up to a 10 mg/mL phospholipid concentration. After the complete dispersion of the lipid films, the obtained mixtures were slowly stirred in a water bath, at the same temperature chosen for the buffer, for about 1 h until the induction of a homogeneous suspension. The dispersions of multilamellar lipid vesicles (MLVs) obtained were extruded through polycarbonate filters (pore size of 100 nm) mounted on a heated miniextruder (Avanti Polar Lipids, Alabaster, AL, USA) fitted with two 1 mL gas-tight syringes (Hamilton, Reno, NV, USA) in order to obtain suspensions of small unilamellar vesicles (SUVs). The extrusions were carried out at 65 °C, i.e., a temperature above the gel-to-liquid-crystal transition of the lipid system (generally a temperature of 10 °C higher than the phase transition temperature of the hardest lipid of the mixture). An odd number of passages, usually 41, was performed to avoid any contamination by liposomes that might have not passed through the filters, as suggested elsewhere.^45^ As for FFAs-containing membranes, the acids were mixed with phospholipids prior to dissolving them in chloroform:methanol 3:1.

According to our previous study,^25^ we demonstrated that the protocol applied for the SUVs preparation produces unilamellar vesicles with a distribution around the nominal provided by the supplier (i.e., 100 nm), as indicated by dynamic light scattering data. Furthermore, deviations in liposome size and polydispersity are not able to influence the micro-DSC thermograms in the case of multicomponent systems. For this reason, this characterization was only partially repeated here. The hydrodynamic diameter was only obtained for the SUVs dispersions addressed to the preparation of nisin-containing samples in order to verify the effect of multiple heating–cooling cycles on liposomes (see Thermal Analysis Measurements). Such liposomal formulations were analyzed at 25 °C through a light-scattering instrument (Zetasizer Nano-ZS, Malvern Panalytical Ltd., Malvern, U.K.) with a final phospholipid concentration of 500 μM. We verified that the multiple heating–cooling cycles did not compromise the integrity of the vesicles (Table S1).

### Thermal Analysis Measurements

Calorimetry was used to determine the stabilities of the membranes with specific reference to transitions of the lipid phases. Micro-DSC was selected as the most suitable technique for liposome investigation.^46^ The instrument used was a Setaram micro DSCIII (Setaram Instrumentation, Caluire, France) operating with 1 mL hermetically closed pans at 0.5 °C/min scanning rate. After the conclusion of the liposomes’ preparation protocol, each dispersion was allowed to anneal for at least 30 min at room temperature before DSC measurement was launched. SUVs samples were diluted up to 3.2 mM phospholipid concentration, also for vesicles which included FFAs.^25,26^ The phospholipid concentration was derived by accurately considering the lipid weight and the dilution volumes at each step of the liposome preparation protocol (the validity of such an approach was assessed in previous works^25,26^).

For nisin-containing samples, nisin-free SUVs dispersions were subjected to four heating–cooling cycles through micro-DSC in order to ensure the achievement of stable lipid phases. Then, adequate amounts of scanned SUVs dispersions and nisin stock solution were mixed and diluted in buffer just before measurements were launched, achieving 30 μM and 3 mM concentrations for nisin and phospholipids, respectively (1:100 nisin:phospholipid ratio). Four heating–cooling cycles were applied to each sample in order to achieve and ensure the reproducibility of the lipid phases.^26^ All transitions were reversible, and the last cycle heating curves were considered to evaluate the parameters of the thermotropic transitions observed (Figures S3 and S4). Errors were evaluated on the basis of at least three replicas.

The raw data were worked out with the dedicated software THESEUS.^47^ Briefly, the apparent specific heat trace, *C*_*p*_^app^(*T*), was scaled to obtain the excess specific heat, *C*_*p*_^exc^(*T*), with respect to the low temperature lipid state. Due to such a treatment, the area beneath the recorded peaks directly corresponded to the relevant transition enthalpy Δ*H*° of the lipid phase.

In order to quantitatively compare and discuss the transition cooperativity between different systems, we adopted the transition average temperature, *T̅*, and the average cooperativity index, ACI, defined elsewhere.^25^ Briefly, the transition average temperature, *T̅*, is defined as
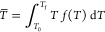
1where *T*_0_ and *T*_f_ are the initial and final limits of the observable peak, respectively, and the frequency function *f*(*T*) is the normalized calorimetric peak distribution
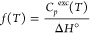
2whereas the average cooperativity index, ACI, is defined as
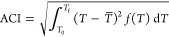
3The higher the ACI value, the lower the transition cooperativity.

### Fluorescence Spectroscopy

Fluorescence anisotropy measurements were performed with a PerkinElmer LS-55 spectrofluorometer (PerkinElmer, Waltham, MA, USA). To monitor the fluidity of phospholipid bilayers, the apolar diphenylhexatriene (DPH) was used as fluorescent probe and was incorporated within the hydrophobic region of the vesicle bilayer.^48^ The excitation and emission wavelengths were 348 and 426 nm, respectively.^49−51^ The bandwidth of the excitation monochromator was 4 nm, whereas the bandwidth of the emission monochromator was 2.5 nm. The fluorescent probe was added up to 1 μM (500:1 phospholipid/fluorescent probe molar ratio). Treated samples were well-mixed and incubated in the dark under continuous stirring for 2 h at 37 °C before measurement. The temperature of the cuvette holder was controlled (Thermo Fisher Scientific, Haake SC 100, Waltham, MA, USA), and temperature scans were performed within the range from 10 to 64 °C with temperature steps of 3 °C, allowing the samples to equilibrate prior to the reading of fluorescence anisotropy values. The fluorescence anisotropy values, *r*, were calculated by the fluorescence data manager program using Jablonski’s equation:
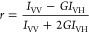
4where *I*_VV_ and *I*_VH_ are the vertical and horizontal fluorescence intensities, respectively, to the vertical polarization of the excitation light beam. The factor *G* = *I*_HV_/*I*_NH_ (grating correction factor) corrects the polarizing effects of the monochromator.

## Results and Discussion

### Model Membrane Design and Characterization

In order to investigate the influence of free fatty acids (FFAs) on peptide–cell membrane interaction, a model unilamellar lipid membrane was designed to fulfill the following criteria:

(a) The first criterion is simple but informative, i.e., with a lipid composition complexity just enough to discriminate the main thermodynamic contributions in terms of the phospholipid headgroup, tails, and presence of unsaturations.

(b) The second criterion is to display a gel-to-liquid-crystalline phase transition throughout a temperature range and with a cooperativity index (dispersion) close to those exhibited by both the high-complexity and real lipid bilayers.^26,40,41^

Considering the hierarchy of contributions that regulate the cell membrane thermodynamics,^25,26^ at the first stage of the design DMPC and DPPS in a 3:2 molar ratio (respectively) were selected as the main phospholipids for vesicle modeling by consideration of the *T*_max_ of the gel-to-liquid-crystalline phase transition of the pure constituents, i.e., *T*_max_ = 24.0 ± 0.2 °C for DMPC^25^ and *T*_max_ ≈ 55 °C for DPPS,^52,53^ as well as the presence of at least two different tails and headgroups.

**Figure 1:**
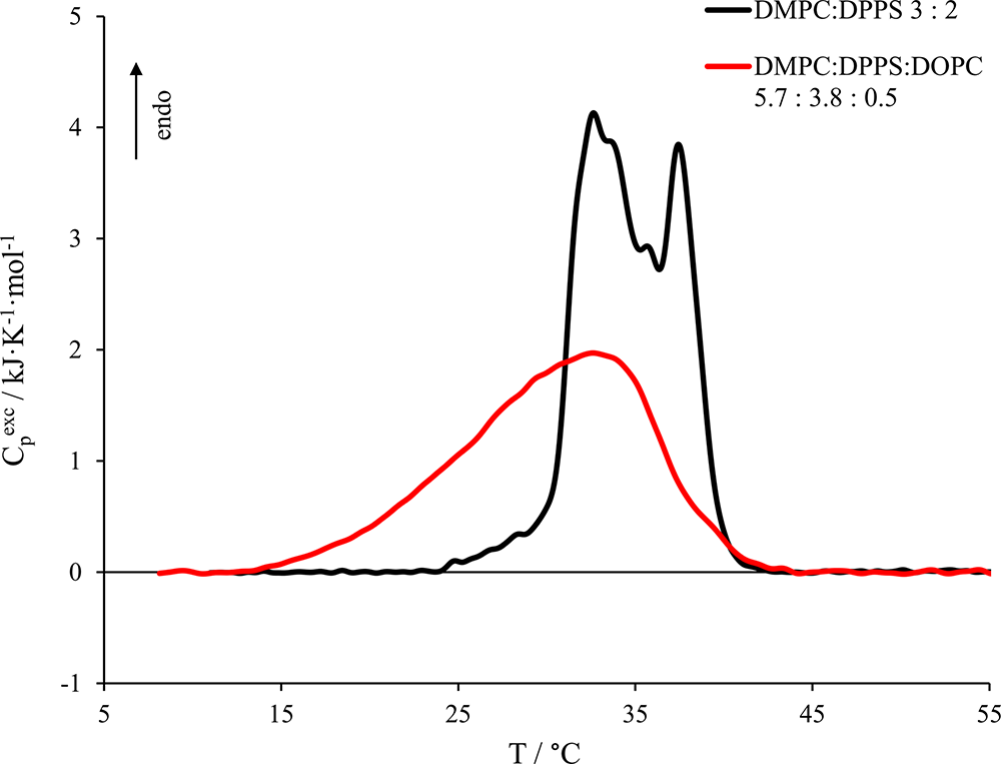
Micro-DSC profiles for DMPC:DPPS 3:2 vesicles (black curve) and vesicles obtained by the addition of 5% DOPC to the DMPC:DPPS 3:2 system achieving a 5.7 DMPC:3.8 DPPS:0.5 DOPC molar ratio (red curve).

The micro-DSC thermogram for such vesicles is reported in Figure 1 as a black trace and corresponds to the main lipid gel-to-liquid-crystalline phase transition. We observe a biphasic profile reflecting a pronounced phase separation due to the low thermodynamic compatibility of the constituents due to the different headgroups and tail lengths. However, as expected for saturated phospholipids,^26^ the profile is located within the temperature range defined by the *T*_max_ of the respective single-component systems, and the transition average temperature, *T̅*, is comparable to the expected value calculated by considering the proportion of the phospholipids, as reported in Table 1. As for the enthalpic contribution to the transition (Table 1), the overall enthalpy observed results are additive compared to those of the single-component systems. Hence, the absence of relevant extra enthalpic contribution once again confirms that the thermotropic behavior of these vesicles is mainly entropically driven and follows a composition-dependent proportionality.^25,26^

**Table 1:** Thermodynamic Parameters Evaluated from Micro-DSC Thermograms Obtained from the Modeling of a Simplified Model Membrane^a^

	expected		experimental			
	Δ *H*° (kJ·mol^–1^)	*T*_expected_ (°C)	Δ*H*° (kJ·mol^–1^)	*T*_max_ (°C)	*T̅* (°C)	ACI (°C)
3:2 DMPC:DPPS	30	36.4	30 ± 2	32.6 ± 0.1	34.5 ± 0.1	2.8 ± 0.1
5.7:3.8:0.5 DMPC:DPPS:DOPC	29	–	26 ± 2	32.6 ± 0.6	30.1 ± 0.1	5.3 ± 0.1

a The parameters were compared with the arithmetical values calculated from single-component systems. The last cycle heating curves were used to obtain the main transition enthalpy (Δ*H*°), the peak maximum temperature (*T*_max_), the transition average temperature (*T̅*), and the average cooperativity index (ACI).

For the final step of the design, 5% DOPC was added to the 3:2 DMPC:DPPS mixture in order to enhance the thermodynamic homogeneousness of the lipid phase as well as to include an unsaturated phospholipid, achieving a 5.7 DMPC:3.8 DPPS:0.5 DOPC molar mixture as the final composition. From here on out, we will refer to such a system as the “model membrane”.

The micro-DSC profile obtained for the model membrane is shown in Figure 1 as a red trace, and the relevant thermodynamic parameters are reported in Table 1. We observe an asymmetric and much broader thermogram than the binary system’s one, as revealed by the marked increase of the ACI value up to 5.3 ± 0.1 °C, and a more homogeneous distribution of the lipid microstate stability as a consequence of the addition of unsaturations. Moreover, the *T̅* is considerably shifted toward lower temperatures (30.1 ± 0.1 °C), whereas the overall transition enthalpy decreases in accordance with the literature.^25,26^

To sum up, the choice of considering DMPC and DPPS as 3:2 molar ratio for the model membrane allowed achievement of vesicles with a reasonable ratio between zwitterionic and negatively charged phospholipids^54^ and with a such tail length difference to produce a significant peak breadth after the addition of only 5% DOPC. Furthermore, the micro-DSC profile obtained for this model membrane exhibits a realistic calorimetric profile in terms of transition cooperativity and enthalpy. Indeed, such a profile is similar to that previously obtained for highly representative 15-component lipid vesicles^26^ and is also similarly placed as the few calorimetric profiles for real cell membranes reported in the literature, which display gel-to-liquid-crystalline phase transitions that cover a temperature range of about 30–35 °C.^40,41^ Nevertheless, we would like to point out that our comparison with the real membranes’ DSC profiles reported in the literature is merely qualitative and indicates that the real membrane peculiarities do not compromise the general conclusions here presented about the membrane thermodynamics and the effects of FFAs. In other words, though the real systems may have very different lipid compositions, their calorimetric profiles do not present details far from those expected by considering other membrane constituents.

This model membrane was used for the investigation of the influence of FFAs on the vesicle–nisin interaction.

### Influence of FFAs Chemical Structures on Lipid Membrane Thermal Stability

**Figure 2:**
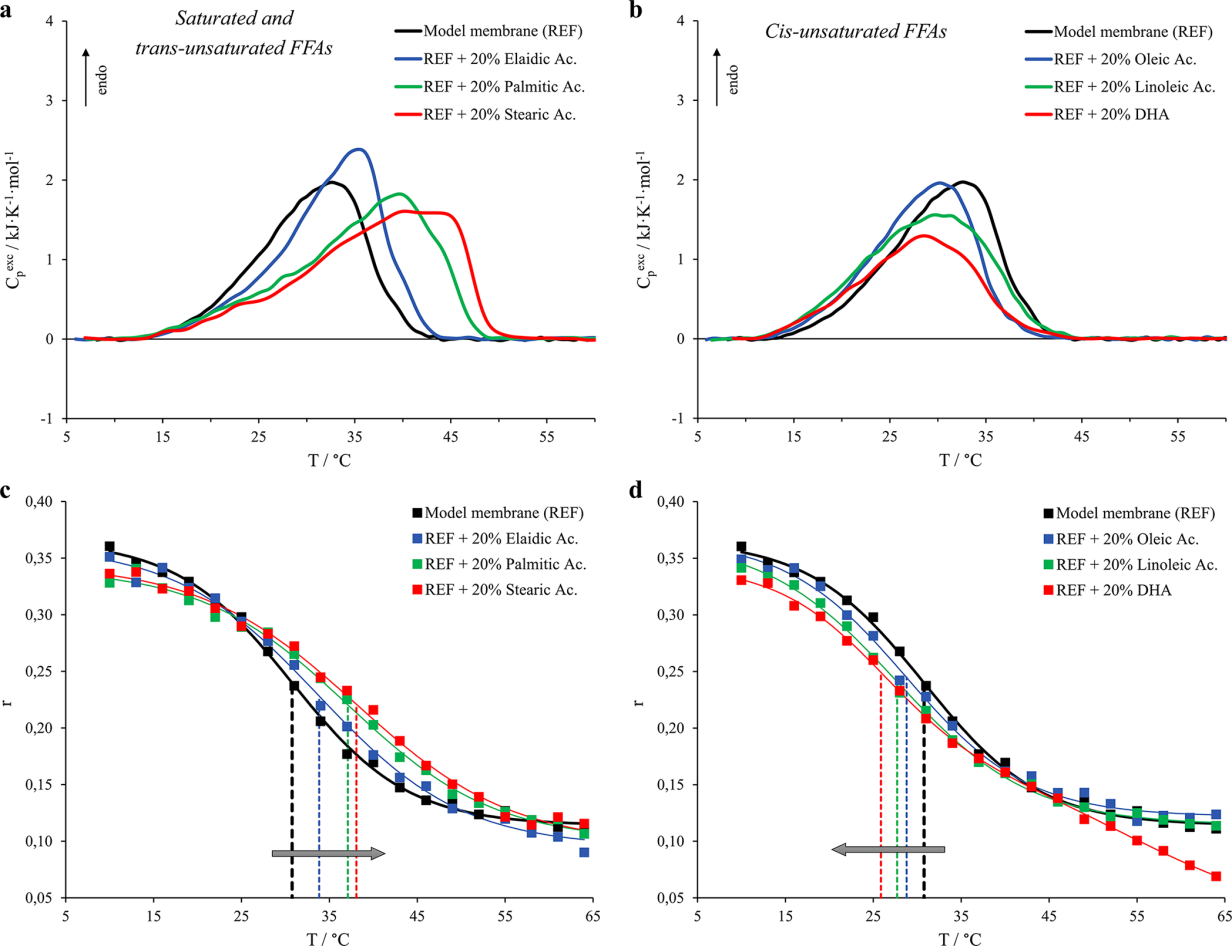
Micro-DSC profiles for model membrane (5.7 DMPC:3.8 DPPS:0.5 DOPC, black curve) and vesicles with the addition of 20% FFAs and corresponding fluorescence anisotropy (*r*) of DPH trapped within the named systems. Thermograms are reported for membranes including saturated and *trans*-unsaturated FFAs in panel a, whereas the *cis*-unsaturated FFAs are shown in panel b. As for the fluorescence anisotropy (*r*) trends, the FFAs-free system is shown as black squares, whereas the FFAs considered are (c) elaidic acid (blue squares), palmitic acid (green squares), and stearic acid (red squares) and (d) oleic acid (blue squares), linoleic acid (green squares), and DHA (red squares). The dashed lines indicate the flex point of the sigmoidal fits with the respective colors. Probe:lipid molar ratio was 1:500.

In order to discriminate the influence that the several FFAs may have on the interaction between the model membrane and nisin, preliminary measurements were performed for the assessment of the effect of the single selected fatty acids on the thermotropic behavior of the model system in relation to their length, level of unsaturation, and/or C=C double bond conformation. The addition of 20% of different FFAs, namely two saturated, a *cis*-monounsaturated, a *trans*-monounsaturated, and two *cis*-polyunsaturated fatty acids, was investigated, and the micro-DSC thermograms and the relevant thermodynamic parameters for vesicles containing the six different acids are hence reported in Figure 2a,b and Table 2, respectively.

**Table 2:** Thermodynamic Parameters Evaluated from Micro-DSC Investigations for Several FFA-Containing Vesicles Alone and in the Presence of 30 μM Nisin^a^

	calorimetry				
	Δ*H*° (kJ·mol^–1^)	*T*_max_ (°C)	*T̅* (°C)	ACI (°C)	spectroscopy *T*_flex_ (°C)
model membrane (REF)	26 ± 2	32.6 ± 0.6	30.1 ± 0.1	5.3 ± 0.1	30.8 ± 0.4
REF + nisin	28 ± 2	34.3 ± 0.5	30.7 ± 0.1	5.6 ± 0.1	–
REF + PA	30 ± 2	39.5 ± 0.4	34.9 ± 0.2	7.3 ± 0.2	37.1 ± 0.6
REF + PA + nisin	35 ± 2	39.3 ± 0.7	34.4 ± 0.2	7.1 ± 0.2	–
REF + SA	30 ± 2	39.9 ± 0.6	36.6 ± 0.2	7.6 ± 0.2	38.1 ± 0.7
REF + SA + nisin	28 ± 2	41.5 ± 0.2	36.6 ± 0.2	7.4 ± 0.2	–
REF + EA	30 ± 2	35.0 ± 0.6	31.9 ± 0.2	5.5 ± 0.2	33.8 ± 0.7
REF + EA + nisin	28 ± 2	36.0 ± 0.2	32.7 ± 0.2	5.7 ± 0.2	–
REF + OA	24 ± 2	30.6 ± 0.6	28.1 ± 0.2	5.1 ± 0.2	28.8 ± 0.6
REF + OA + nisin	27 ± 2	29.6 ± 0.5	29.1 ± 0.2	5.7 ± 0.2	–
REF + LA	25 ± 2	29.9 ± 0.4	28.5 ± 0.2	6.1 ± 0.2	27.7 ± 0.5
REF + LA + nisin	20 ± 2	26.8 ± 0.5	25.8 ± 0.2	5.7 ± 0.2	–
REF + DHA	18 ± 2	28.3 ± 0.9	27.6 ± 0.2	5.9 ± 0.2	25.9 ± 1.5
REF + DHA + nisin	29 ± 2	32.0 ± 0.5	29.3 ± 0.2	5.9 ± 0.2	–

a The last cycle heating curves were used to obtain the main transition enthalpy (Δ*H*°), the peak maximum temperature (*T*_max_), the transition average temperature (*T̅*), and the average cooperativity index (ACI). The transition average temperature (*T̅*) was also compared to the temperature at the sigmoid flex point (*T*_flex_) obtained from fluorescence spectroscopy measurements. The abbreviations in the table indicate palmitic acid (PA), stearic acid (SA), elaidic acid (EA), oleic acid (OA), linoleic acid (LA), and docosahexaenoic acid (DHA).

We observe that the addition of 20% palmitic and stearic acids, i.e., the two saturated fatty acids (16:0 and 18:0, respectively), generates a more severe membrane stabilization in terms of *T̅* and a more considerable loss of cooperativity (increase of ACI values) if compared to the effect deriving from elaidic acid (18:1Δ^9t^), in accordance with the results already reported in the literature for more complex systems.^25,26^ Nevertheless, the enthalpic stabilizations with respect to the FFAs-free model membrane in this case are of the same order for all three systems containing the FFAs mentioned above. On the other hand, the incorporation of 20% of all the *cis*-unsaturated FFAs leads to a moderate decrease of the membrane thermodynamic stability and the magnitude of their impact depends on the number of C=C double bonds (Table 2). The micro-DSC thermograms indicate that their destabilizing effect is more pronounced on the high-stability lipid phases. Specifically, linoleic acid (18:2Δ^9c,12c^) reduces the transition cooperativity more than oleic acid (18:1Δ^9c^) as revealed by the ACI value, whereas the high irregularity of DHA’s molecular structure (22:6Δ^14c,7c,10c,13c,16c,19c^) is mainly reflected in a strong decrease of the enthalpic contribution to the gel-to-liquid-crystalline phase transition.

Complementary information about the influence of the different FFAs on the membrane lipid organization was also obtained by fluorescence spectroscopy. The sigmoidal trends of the DPH’s fluorescence anisotropy values (*r*) against the temperature obtained for the model membranes containing saturated and *trans*-unsaturated FFAs are reported in Figure 2c, whereas Figure 2d shows the trends for vesicles containing the *cis*-unsaturated FFAs.

We observe that the sigmoids for vesicles containing stearic and palmitic acids are shifted toward higher temperatures than that for elaidic acid, while the ones for vesicles containing oleic acid, linoleic acid, and DHA are increasingly shifted toward lower temperatures depending on the number of unsaturated C=C bonds. The DPH’s fluorescence anisotropy value reflects the average local levels of order and packing of phospholipid acyl chains, which decrease as a function of the temperature as the main lipid gel-to-liquid-crystalline phase transition proceeds. In analogy with the DSC results reported in Figure 2a,b and those widely discussed in a previous work,^25^ we may assess that stearic, palmitic, and elaidic acids are able to fit into the phospholipid–phospholipid intermolecular space because of their linear shapes, increasing the vesicle molecular packing. By contrast, “angled” *cis*-fatty acids disturb the intermolecular order with an extent of the effects that depends on the level of unsaturation of the acyl chains.

For a better comparison, for this work we also consider the flex point of the sigmoids, which should approximately correspond to a 50% average degree of advancement of the process and, thus, may be compared with the average temperature of the transition *T̅* detected by the DSC (Figures S5 and S6). A comparison between the *T̅* values from micro-DSC curves and the flex point temperature of the sigmoids from DPH’s fluorescence anisotropy obtained for the model membrane alone and containing the 20% of various FFAs is reported in Table 2. We observe that the flex point temperatures obtained from the spectroscopic experiments are in substantial accordance with the *T̅* values of the calorimetric ones, confirming once again the type of structural effect produced by FFAs with different molecular geometries well reflects the effects on the membrane thermodynamic stability (slight differences may be ascribable to the resolution of the anisotropy readings and to the few points obtained by the discrete heating ramp with 3 °C steps).

Conclusively, the peculiar effects of FFAs on the phospholipid bilayer of the model system, based on their chemical structures, can be considered as reference for the detection of the possible FFA–nisin combined action on the model membrane in each case.

### Nisin–Membrane Interaction

**Figure 3:**
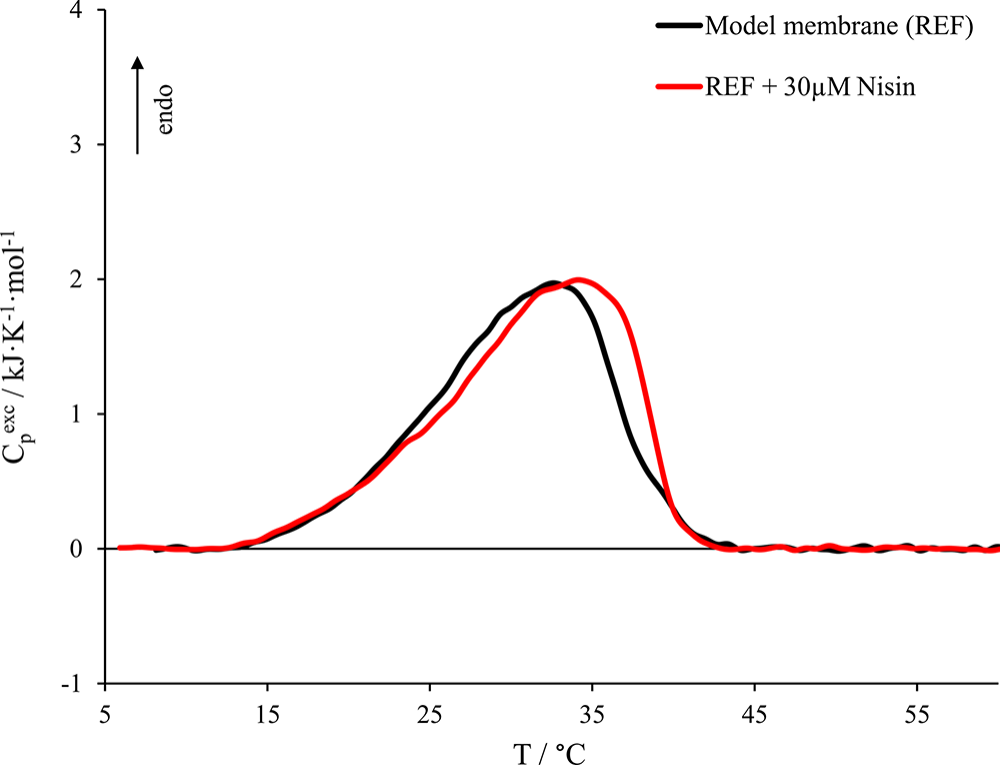
Micro-DSC profile in the presence of 30 μM nisin (red curve). Nisin:phospholipid molar ratio was 1:100. The model membrane alone (black curve) is also reported here for comparison.

Figure 3 shows the comparison of micro-DSC thermograms obtained for the model membrane (5.7 DMPC:3.8 DPPS:0.5 DOPC) alone and in the presence of 30 μM nisin, whereas the relevant thermodynamic parameters are reported in Table 2.

We observe that the presence of 30 μM nisin, which corresponds to a nisin:phospholipid molar ratio of 1:100, leads to a moderate stabilizing effect from both an enthalpic and an entropic point of view, though the transition covers the same temperature range as without peptide. This stabilization is more pronounced in the high-stability lipid phases, indicating that the approach and/or insertion of the peptide within the bilayer is able to enhance thermodynamic phase separations as well as a general lipid reorganization, as also suggested by the literature.^38^ Indeed, considering the composition of the model membrane, the high-stability region corresponds to lipid phases rich in DPPS (*T*_max_ ≈ 55 °C),^52,53^ a negatively charged phospholipid. We may argue that when the positively charged nisin approaches the outer leaflet of the vesicle, the peptide recruits DPPS molecules generating a local higher concentration of these lipids in its surrounding area on the basis of the electrostatic attraction, as also usually happens in the presence of bivalent cations.^55^ The recruitment of DPPS molecules is also supported by the slight enthalpic stabilization, a symptom of the increase of the number of intermolecular interactions between palmitoyl–palmitoyl chains (DPPS–DPPS) if compared to the interactions formed between palmitoyl–myristoyl chains (DPPS–DMPC). Moreover, the DPPS headgroup (serine) is smaller than the choline constituting the DMPC. The truncated conical shape of DPPS might promote the approaching or a partial insertion of the hydrophobic portion of the peptide within the hydrophobic region of the vesicles, which may enhance the peptide–vesicle interaction. Nevertheless, we would like to point out that, though the micro-DSC analysis clearly reveals the interaction of the peptide with the vesicles, it cannot distinguish between the simple peptide approach and its insertion within the liposome hydrophobic region. We may only argue that both the approach and at least a partial insertion are compatible with our experimental conditions.^38,39^

For this work we selected a nisin:phospholipid molar ratio of 1:100 in order to avoid or at least mitigate side effects due to high nisin concentration. Indeed, higher nisin/phospholipid ratios (>5:100) than the one considered in this work are even supposed to strongly interact with the phospholipid bilayer enough to induce pore formation with possible membrane destruction, as reported in the literature.^38,39^

In conclusion, our data confirm that nisin interacts directly with the FFAs-free membrane producing, at low peptide/phospholipid ratios, a moderate stabilizing effect more pronounced in the high-stability lipid phases, which are rich in the negatively charged phospholipid.

**Figure 4:**
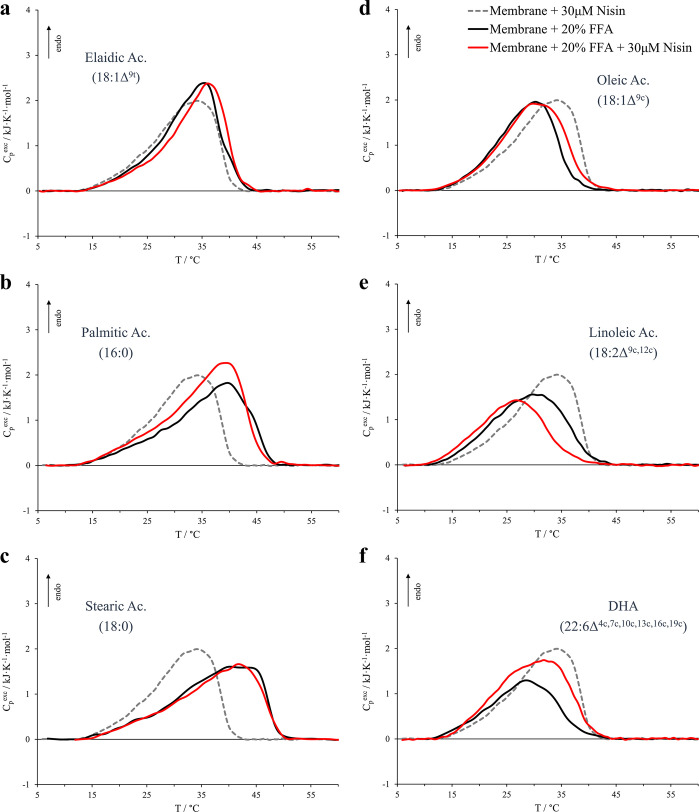
Micro-DSC profiles for model membrane containing 20% different FFAs (black curves) and for the same vesicles in the presence of 30 μM nisin (red curves). The FFAs considered are (a) elaidic acid, (b) palmitic acid, (c) stearic acid, (d) oleic acid, (e) linoleic acid, and (f) docosahexaenoic acid (DHA). The profile of the FFAs-free membrane in the presence of 30 μM nisin is also represented for comparison by the dashed gray trace. Nisin:phospholipid molar ratio was 1:100.

The influence of nisin (nisin:phospholipid molar ratio of 1:100) on the various vesicles containing the FFAs is reported in Figure 4. At a first glance, we observe that all the systems undergo a lipid reorganization upon interacting with the peptide. The different vesicles exhibit peculiar modifications in the gel-to-liquid-crystalline phase transitions that depend on the nature of the incorporated fatty acid. The model membranes containing palmitic, oleic, and DHA acids are characterized by substantial entropic and enthalpic stabilizations of the transition. The model membrane containing elaidic acid is only slightly entropically stabilized, whereas the membrane containing stearic acid is the least affected by nisin in terms of both entropic and enthalpic contributions, as revealed by *T̅*, ACI, and Δ*H*° values in Table 2. On the other hand, vesicles containing linoleic acid exhibit a severe destabilization from both enthalpic and entropic points of view.

In an attempt to categorize the thermotropic behavior of such systems, we may divide the description into two parts.

As far as the saturated and *trans*-unsaturated FFAs are concerned (left side in Figure 4), we are in the presence of membranes that are already stabilized by the FFAs action and the nisin produces only a slight further stabilization. The largest enthalpic stabilization exceptionally observed for vesicles containing palmitic acid may be ascribed to the peculiar fact that this last system is the only one that contains FFAs molecules with an acyl chain that perfectly matches the tail length of one of the constituents of the membrane, i.e. DPPS tails. As already reported in the literature, increasing amounts of palmitic acid in membranes containing a high percentage of dipalmitoyl phospholipids lead to both an entropic and enthalpic stabilization of the systems.^25^ Therefore, we may argue that, in our case, a segregation of palmitic acid within DPPS-rich regions is promoted because of the DPPS recruitment realized by nisin, thus enhancing lipid chains interactions and increasing the enthalpic contribution to the phase transition.

As regards the vesicles containing *cis*-unsaturated FFAs (right side in Figure 4), we are in the presence of membranes that are destabilized by the FFAs action and the nisin stabilizing effect is more evident with the exception of the linoleic system. As for the oleic acid system, we may argue that the DPPS reorganization induced by nisin segregates the fatty acid molecules and some of the tail–tail interactions are hence partially recovered. In the case of DHA, since the stability of the initial system was severely compromised by the presence of DHA’s molecular irregularity due to the six *cis* double bonds, the nisin stabilizing effects seem to be more pronounced. Indeed, as indicated by the micro-DSC profile and by the enthalpy values (red curve in Figure 4f and Table 2, respectively), these stabilizing effects are strongly reflected in the enthalpic contribution to the transition and, moreover, they also involve the low-stability phospholipid phases.

Despite the peculiarities, all these scenarios seem again coherent with a nisin stabilizing effect due to promotion of thermodynamic lipid phase separations, mitigating the destabilizing effect of *cis*-unsaturated FFAs.

On the other hand, as regards the system that contains linoleic acid, this pattern is no longer valid and the nisin promotes a further destabilization of the system both from an enthalpic point of view and from an entropic point of view. On the basis of the DSC profile (red trace in Figure 4e), again we observe a major effect in the high-stability lipid phases (DPPS-rich phases). At this stage, our data are not sufficient to interpret this peculiar behavior and a deeper and specific investigation is required.

Nevertheless, besides our structural interpretations that may require further investigations that are beyond the scope of this paper, the overall thermodynamic picture clearly indicates that the presence of FFAs influence the peptide–membrane interaction in a peculiar manner. In other words, our data highlight how the interaction between cell membranes and proteins/peptides does not overlook the presence of FFAs within the lipid bilayer and the magnitude of the effects may be different depending on the FFAs chemical structure and in turn may affect the biological aspects. Moreover, such an interaction is not able to leave the composition of the phospholipid bilayers out of consideration, as well as any possible compositional modification in the cell membrane. Indeed, as indicated by previous works, we remind the reader here that, although the type of the effects may be recognized, their magnitude is dependent on the phospholipid bilayer composition.^25,26^

## Conclusions

The preparation of a simple but informative model membrane with the presence of different phospholipid headgroups in charge and size (choline and serine) and different phospholipid tails in length and unsaturation level (myristoyl, palmitoyl, and oleoyl chains) allowed the discrimination of the combined interaction of nisin and FFAs with the single phospholipid constituents.

The action of 1:100 nisin:phospholipid ratio on the FFAs-free model membrane revealed a more pronounced membrane stabilization in the high-stability lipid phases, suggesting a preferable interaction of the peptide with the negatively charged phospholipid and the possible partial insertion of the hydrophobic portion of the peptide within the hydrophobic region of the vesicles which might be encouraged by the presence of a smaller headgroup (serine compared to choline). This stabilization involves both the enthalpic and entropic contributions, indicating that the approach of peptide within the bilayer is able to enhance thermodynamic phase separations as well as a general lipid reorganization, as is also suggested by the literature.^38^

The presence of FFAs strongly modifies the model membrane stability in a peculiar manner and, in turn, influences the nisin–vesicle interaction. In particular, the presence of saturated and *trans*-unsaturated FFAs produces stabilization effects on the model membrane and the interaction with nisin results only in a slight further stabilization. On the other hand, the action of *cis*-unsaturated fatty acids (oleic, linoleic, and docosahexaenoic acids) produces overall destabilizing effects to the model membrane whose magnitude increases with the number of C=C double bonds. In this case, the interaction of nisin with the vesicle depends on the FFAs since it is stabilizing for the oleic and DHA systems, while it is destabilizing for the linoleic one.

We may conclude that the peptide–membrane interaction does not overlook the presence of FFAs within the lipid bilayer since both FFAs and nisin are able to selectively enhance thermodynamic phase separations as well as a general lipid reorganization within the host membrane, and the magnitude of the effects may be different depending on the FFAs chemical structure as well as the membrane lipid composition.

Although this study considers a specific peptide (nisin), the overall thermodynamic picture suggests that the conclusions may be extended to other peptides and proteins that are able to enhance a thermodynamic phase separation within the lipid bilayers. Accordingly, when in the presence of FFAs, their chemical nature has to be considered in order to interpret the influence on such peptide–membrane interactions, which in turn may influence the biological aspects of the system.

## Supporting Information Available

Details on nisin purification; details on micro-DSC profiles about DMPC:DPPS 3:2 vesicles (third and fourth heating scans); micro-DSC thermograms showing reversibility of lipid vesicles’ gel-to-liquid-crystalline phase transition; DLS parameters on scanned SUV dispersions; comparison between fluorescence anisotropy of DPH in model vesicles containing 20% linoleic acid and respective micro-DSC profile; comparison between transition average temperatures and the sigmoids’ flex point obtained from calorimetric and spectroscopic investigations (PDF)
