# Supplementary material for: Influence of Free Fatty Acids on Lipid Membrane–Nisin Interaction
Source: Langmuir. 2020 Nov 2;36(45):13535–44. doi: 10.1021/acs.langmuir.0c02266 (PMC8016202; doi:10.1021/acs.langmuir.0c02266)
Supplement: Supplementary file 1 — la0c02266_si_001.pdf [file la0c02266_si_001.pdf]

Supporting Information

Influence of free fatty acids on lipid membrane-nisin interaction

Francesca Saitta[a], Paolo Motta[a], Alberto Barbiroli[a], Marco Signorelli[a], Carmelo La Rosa[c], Anna Janaszewska[b], Barbara Klajnert-Maculewicz[b] and Dimitrios Fessas[a,*]

[a] Dipartimento di Scienze per gli Alimenti, la Nutrizione e l'Ambiente, DeFENS, Università degli Studi di Milano, Via Celoria 2, 20133, Milano, Italy

[b] Department of General Biophysics, Faculty of Biology and Environmental Protection, University of Lodz, 141/143 Pomorska St., 90-236 Lodz, Poland

[c] Dipartimento di Scienze Chimiche, Università degli Studi di Catania, Viale Andrea Doria 6, 95125, Catania, Italy

*Corresponding author. E-mail address: dimitrios.fessas@unimi.it. Tel.: +39 0250319219

Number of pages: 7

Number of figures: 6

Number of tables: 1

Table Of Contents

EXPERIMENTAL SECTION S2
Nisin purification S2
Thermal analysis measurements S3
Dynamic Light Scattering S5
RESULTS AND DISCUSSION S6
Influence of FFAs chemical structure on lipid membranes thermal stability S6

EXPERIMENTAL SECTION

Nisin purification

Figure S1 shows the SDS-PAGE gel electrophoresis depicting the purification of nisin. As revealed by the first three lanes at different nisin concentrations, the purification protocol applied to the commercial powder provided a highly purified peptide (no peptides other than the nisin band at about 3.5 kDa are visible in the gel). Such a result was supported by both the last three lanes, which were performed at high nisin concentrations in order to better detect any possible contaminant. HPLC chromatogram reported in Figure S2 quantified the purity grade of protein, which was >95% as reported in the experimental section.

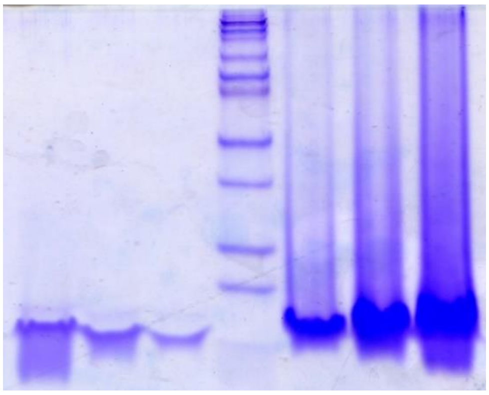

Figure S1. *SDS-PAGE gel electrophoresis for the purified nisin. The first three lanes and the last three ones correspond to nisin solutions at different concentrations.*

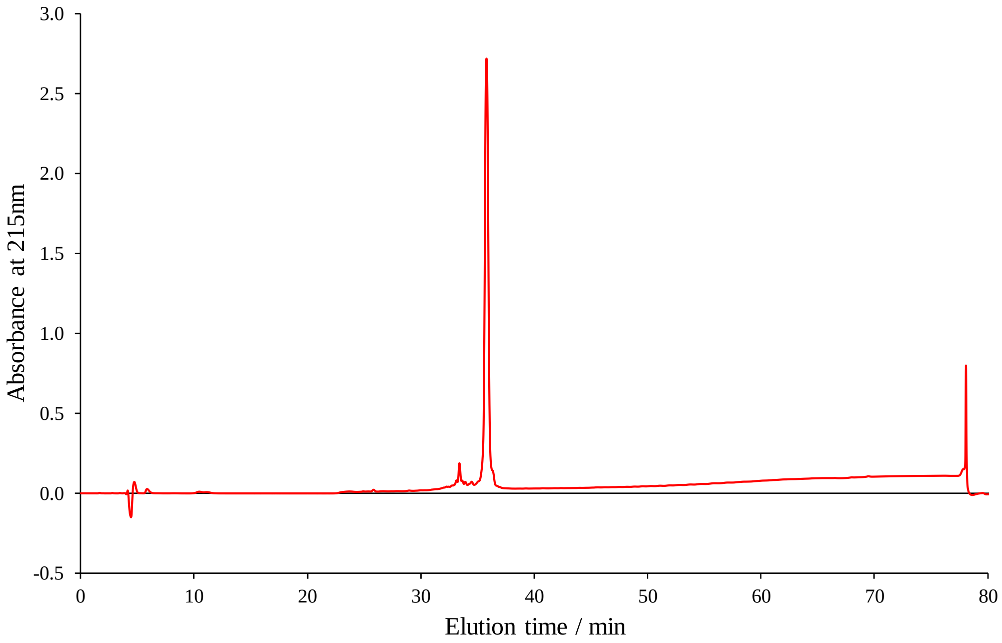

Figure S2. *HPLC chromatogram for the purified nisin.*

Thermal analysis measurements

Generally, the application of two heating/cooling cycles to vesicle dispersions is enough for the achievement of equilibrium phases, allowing the use of the second heating/cooling cycle for the analysis. In other words, such cycle may be indicated as the thermodynamically meaningful one since any other following cycle would lead to almost the same calorimetric profile.

However, lipid phases in metastable equilibria and kinetic phenomena may arise if the phospholipid constituents do not manifest a good thermodynamic compatibility. In this case, more heating/cooling cycles might be necessary.

As far as the DMPC:DPPS 3:2 system is concerned, the application of the liposome preparation protocol allowed a good mixing of the constituent, so much to obtain a micro-DSC thermogram with a well dispersed and quite homogenous calorimetric profile at the first heating ramp. However, the lipid phases started to segregate because of the low thermodynamic compatibility of the constituents from the next (second) heating scan. The micro-DSC thermograms deriving from the application of the third and fourth heating/cooling cycles to this binary membrane are shown in Figure S3. We observe that the fourth heating scan (reported as a dashed black trace and corresponding to the black curve reported in Figure 1 of the main text) is perfectly superimposable to the third heating scan (reported as a solid red trace), revealing the achievement of a phase equilibrium. The fourth heating scan was considered as thermodynamically meaningful, analogously to what was done for all the other systems considered in this work.

Figure S4 reports the calorimetric profiles obtained for the model membrane (5.7 DMPC : 3.8 DPPS : 0.5 DOPC molar ratio) for the application of a heating/cooling cycle as an example. We observe reversible gel-to-liquid crystalline phase transitions as revealed by the exothermic trace due to the cooling scan (dashed curve). A similar behavior is observed for all the systems considered in this work.

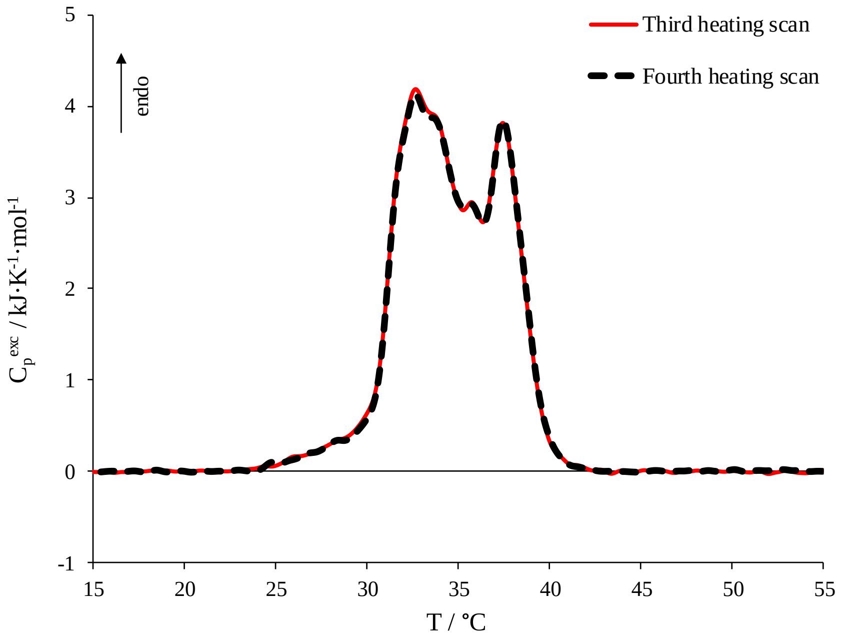

Figure S3. *Micro-DSC profiles for DMPC:DPPS 3:2 vesicles obtained at the application of the third (solid red curve) and fourth (dashed black curve) heating ramps.*

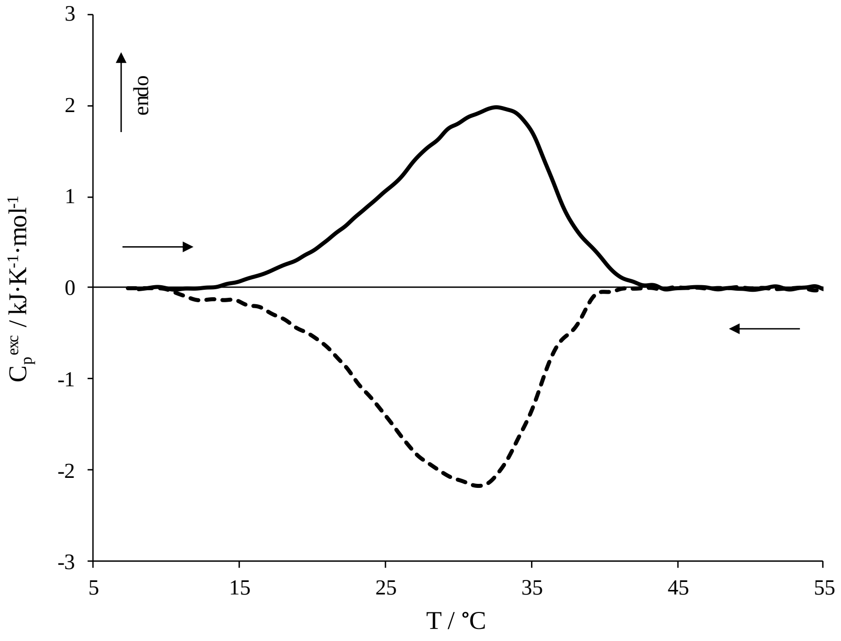

Figure S4. *Micro-DSC profiles for vesicles obtained as a 5.7 DMPC : 3.8 DPPS : 0.5 DOPC molar ratio. The thermograms correspond to the fourth heating and cooling scans (solid and dashed traces, respectively).*

Dynamic Light Scattering

Table S1 shows the data obtained from the DLS analysis on vesicles containing 20% of FFAs and that had already undergone four heating/cooling cycles through micro-DSC in order to verify that the integrity of the vesicles was not compromised. Such scanned SUVs dispersions were addressed to the preparation of nisin-containing samples. As shown in the table, the liposome size resulted to be affected by the application of multiple heating/cooling cycles depending on the type of FFA incorporated within the bilayer. Indeed, unlike the vesicles containing unsaturated FFAs which remained unaffected, we hypothesise that a limited fraction of the liposomes containing saturated FFAs underwent fusion, leading to a considerable increase of the PDIs (we can exclude aggregation or other severe damages because they would have been visible and identified on the micro-DSC thermograms as they would have produced very different profiles than the unilamellar vesicles). Nevertheless, the revealed modifications in vesicle size and PDI are unable to produce modifications in the micro-DSC thermograms, as reported in the literature for multicomponent systems (Saitta, F. *et al.* Colloids and Surfaces B: Biointerfaces 176 (2019) 167–175).

Table S1. *Physicochemical characteristics obtained from DLS measurements for several vesicles containing 20% of FFAs. The reported parameters are z-Average diameter (Z_{ave}) with standard deviation (SD^1) and the polydispersity index (PDI) with standard deviation (SD^2). The labels reported on the table indicate palmitic acid (PA), stearic acid (SA), elaidic acid (EA), oleic acid (OA), linoleic acid (LA) and docosahexaenoic acid (DHA).*

	**Z_{ave} nm**	**SD^1 nm**	**PDI**	**SD^2**
Model membrane (REF)	84.2	0.6	0.279	0.007
REF + PA	103.0	0.9	0.444	0.010
REF + SA	126.9	1.2	0.588	0.011
REF + EA	87.5	0.9	0.325	0.002
REF + OA	67.4	0.2	0.103	0.005
REF + LA	69.4	0.2	0.074	0.004
REF + DHA	75.2	0.1	0.070	0.028

RESULTS AND DISCUSSION

Influence of FFAs chemical structure on lipid membranes thermal stability

Figure S5 shows, as an example, the micro-DSC gel-to-liquid crystalline phase transition obtained for linoleic acid and the superimposition of the sigmoidal trend of fluorescence anisotropy values, *r*, against temperature. We observed that the fall of the anisotropy values well matched the temperature region of the phase transition since the probe's fluorescence anisotropy reflects the levels of order and packing of phospholipid acyl chains. Moreover, the sigmoid flex point was comparable to the $\bar{T}$ obtained from the calorimetric curve. Indeed, the flex point should theoretically correspond to a 50% degree of advancement of the process, thus indicating an average temperature of the transition.

Figure S6 reports a comparison between the $\bar{T}$ values from micro-DSC curves (dotted blue bars) and the flex point of the sigmoids from fluorescence anisotropy (lined red bars) obtained for the model membrane alone and containing the 20% of various FFAs. As already mentioned in the main text, the flex points obtained from spectroscopic experiments were in accordance with the $\bar{T}$ values of the calorimetric ones, confirming once again the type of effect produced by FFAs with different chemical structure. The slight differences between the two values may be ascribable to the few spectroscopic experimental point and to the lower sensitivity of the spectroscopic technique for the detection of lipid phase transitions than the calorimetric one, made also worse by the experimental conditions since the heating ramp for fluorescence measurements was obtained by applying discrete fixed temperature steps of 3°C each.

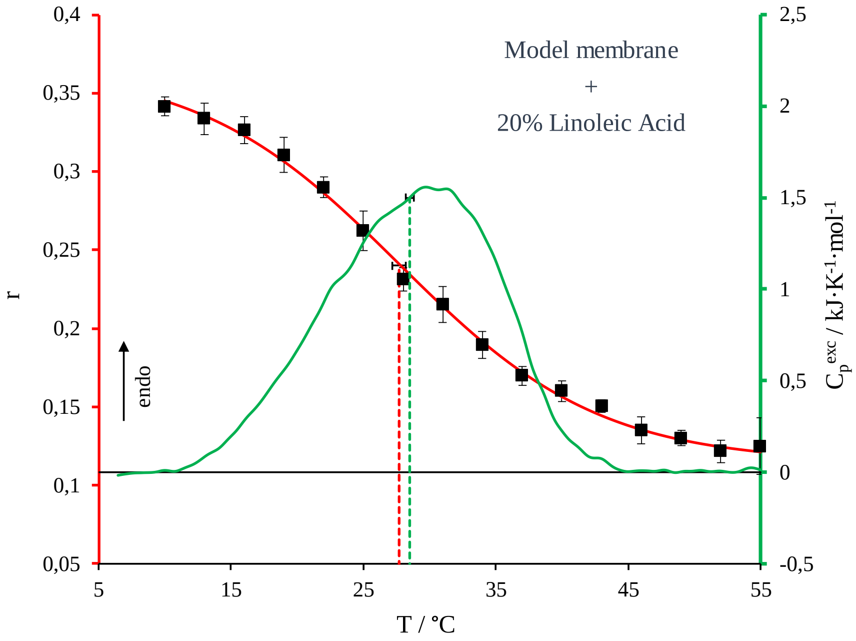

Figure S5. *Example of superimposition of fluorescence anisotropy (r) of DPH in model vesicles containing 20% of linoleic acid (red trace) to the respective micro-DSC profile (green trace). The dashed lines indicate the $\bar{T}$ for the calorimetric curve (green line) and the flex point of the sigmoid obtained from fluorescence anisotropy (red line). Probe:lipid molar ratio was 1:500.*

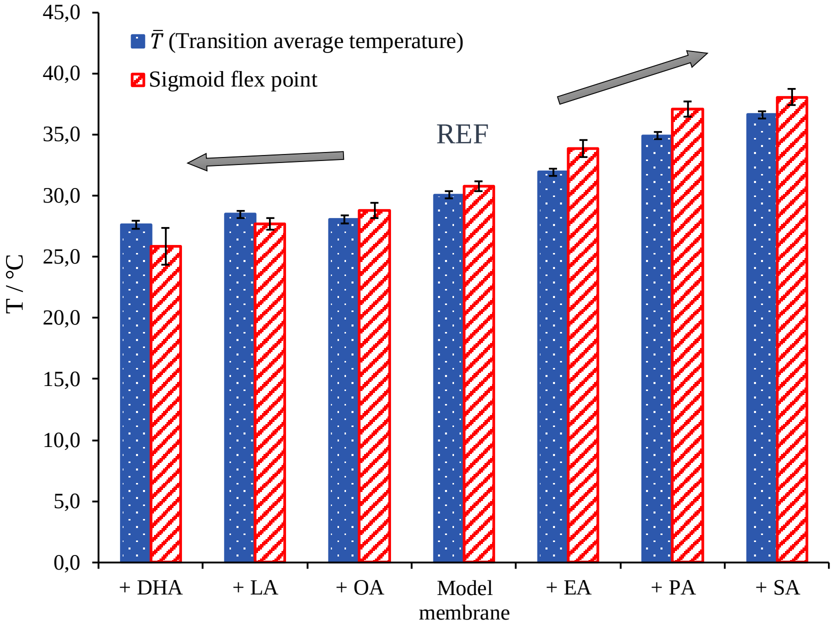

Figure S6. *Histogram representation showing the $\bar{T}$ values from micro-DSC curves (dotted blue bars) and the flex point of the sigmoids from fluorescence anisotropy (lined red bars) obtained for the model membrane alone and containing the 20% of various FFAs. The labels reported on the histogram indicate docosahexaenoic acid (DHA), linoleic acid (LA), oleic acid (OA), elaidic acid (EA), palmitic acid (PA) and stearic acid (SA).*
